# Food Addiction: Prevalence, Severity, and Impact on Vascular Stroke Risk Factors in a French Hospital-Based Sample

**DOI:** 10.3390/nu16244327

**Published:** 2024-12-15

**Authors:** Bibi Aliya Seelarbokus, Yolaine Rabat, Christophe Lalanne, Igor Sibon, Sylvie Berthoz

**Affiliations:** 1 Univ. Bordeaux, Institute for Cognitive and Integrative Neuroscience Aquitaine (INCIA), French National Centre for Scientific Research (CNRS), UMR5287, 33000 Bordeaux, France; bibi-aliya.aliya@u-bordeaux.fr (B.A.S.); yolaine.rabat@hotmail.fr (Y.R.); sylvie.berthoz-landron@inserm.fr (S.B.); 2 University Paris Cité, 75006 Paris, France; christophe.lalanne@aliquote.org; 3 Neurovascular Unit, Bordeaux University Hospital, 33000 Bordeaux, France; 4 Department of Psychiatry for Adolescents and Young Adults, Institut Mutualiste Montsouris, 75014 Paris, France

**Keywords:** food addiction, stroke, hypertension, obesity, diabetes, dyslipidaemia, disordered eating behaviour

## Abstract

Background: Stroke ranks as the second leading cause of death and the third leading cause of disability in adults worldwide. While an unhealthy diet is an independent risk factor for stroke, its association with disordered eating behaviours on stroke remains overlooked. This exploratory study aimed to evaluate the prevalence and severity of addictive-like eating behaviours in stroke patients and their association with the main vascular stroke risk factors. Methods: First-ever minor or moderate stroke patients with the ability to complete self-report questionnaires were included. Addictive-like eating was assessed using the Yale Food Addiction Scale 2.0 (YFAS 2.0). The variables of interest were: (i) the proportion of patients meeting the diagnosis of food addiction (FA); (ii) FA symptoms count and severity; (iii) addictive-like eating profile severity. Their association with four main vascular risk factors (obesity, hypertension, dyslipidemia and diabetes) were tested using univariate and multivariate analyses. Results: Over a 4-month period, 101 patients (mean (standard deviation (SD)), 62.8 (13.7) years; males: 60.4%) were consecutively screened with the YFAS 2.0. Overall, 5% of the sample endorsed an FA diagnosis, and 38.6% screened positive for at least one of the symptom criteria, with the most frequently endorsed symptom being “Inability to cut down”. Dyslipidemia was the only vascular risk factor associated with the FA diagnosis (*p* = 0.043, 95% CI [−0.21; 0.01]). However, the severity of the addictive profile was associated with dyslipidemia (*p* = 0.016, 95% CI [−2.16; −0.21]) and diabetes (*p* = 0.038, 95% CI [−1.77; 0.25]), but only independently with dyslipidemia (*p* = 0.05; OR = 1.25; 95% CI [1.00; 1.56]). There were significant associations between the number of vascular risk factors and the severity of Time spent, Tolerance, and Use despite adverse consequences of FA symptoms, both in univariate and multivariate analyses (all *p* < 0.05). The number of vascular risk factors and total number of FA symptoms were significantly associated in univariate analyses (*p* = 0.007) but not after adjusting for age (*p* = 0.055) or sex (*p* = 0.083). Conclusions: This study highlighted the potential importance of addictive-like behaviours in the secondary prevention of stroke. However, larger and longer-term studies investigating addictive-like eating in diverse samples of stroke patients are warranted to achieve precision medicine.

## 1. Introduction

According to recent Global Burden of Disease (GBD) estimates, stroke is the second leading cause of death and the third leading cause of disability worldwide, with 12.2 million incident cases of stroke, 143 million disability-adjusted life-years (DALYs) lost, and 6.6 million deaths reported in 2019 [1]. Globally, although life expectancy (LE) increased to 73.2 years in 2021, ageing remains a primary risk factor for stroke events, alongside a ratio of healthy life expectancy (HLE) to LE of 0.83 [2]. On the current trajectory, the average age for a first stroke onset is approximately 70 years, coupled with a reduction in case fatality after stroke [3,4]; the demographic shift is expected to significantly increase the prevalence of stroke by 2030 [5]. Considering the distress caused to patients and families, in addition to the healthcare and social costs [6], the implementation of public health action plans seeking to redress the imbalance of healthcare expenses concentrated towards the last stages of life represents a research priority.

Traditional strategies to curb healthcare costs associated with stroke have predominantly focused on compensatory approaches aiming to alleviate the consequences of impairments instead of tackling their causal factors [7]. Nevertheless, with an enhanced understanding of the pathophysiology and aetiology of the disease, there is robust evidence indicating that modifiable risk factors account for 90% of the risk of stroke [8,9,10,11] and can be grouped into specific categories such as diseases (e.g., atrial fibrillation, carotid stenosis, sickle cell disease), physiological factors (e.g., hypertension, high fasting glucose, high body mass index (BMI), high cholesterol, low glomerular filtration rate, chronic inflammation), environmental factors (e.g., air pollution), psychosocial factors (e.g., depression and anxiety, psychosocial stress, job strain, long working hours), and poor lifestyle habits (e.g., dietary items at risk, tobacco, alcohol, and illicit drug abuse, and low physical activity) [12,13]. As the risk of stroke recurrence is highly dependent on the control of these risk factors, the aetiological examination and management of stroke patients should, thus, aim at identifying all potentially modifiable risk factors, as well as their determinants, to provide individualised recommendations for optimal secondary stroke prevention, as promoted in the Stroke Action Plan for Europe (SAP-E) 2018–2020 [12,14].

Of note, whilst dietary risks accounted for 11 million diet-related deaths in 2017, of which cardiovascular diseases were the leading cause worldwide [15], the extant literature has primarily focused on dietary patterns such as the Mediterranean diet, which has been heralded as one of the healthiest patterns exhibiting cardioprotective properties [16,17,18,19]. However, amidst the coincidental prominence of obesogenic environments promoting the intake of palatable foods rich in fat, salt, and sugar with high intrinsic addictive properties, there is emerging evidence to now recognise addictive-like eating behaviours as a potentially major, yet understudied, concerns in patients with stroke [20,21].

In a retrospective World Health Organisation (WHO) study examining the impact of mental disorders (based on the Diagnostic and Statistical Manual of Mental Disorders (DSM) IV-TR) on the risk of stroke that included general population surveys in 17 countries (*n* = 87,250 adults), bulimia nervosa was the mental disorder associated with the highest Odds Ratio (OR) among all disorders evaluated (OR = 3.8; 95% CI: 1.6–9.1), including the abuse or dependence on alcohol or drugs (OR = 2.4; 95% CI [1.5; 3.8] and OR = 2.4; 95% CI [1.1; 5.1], respectively), independently of sociodemographic variables and smoking status [22]. While bulimia nervosa and binge eating disorder are syndromic entities characterised by severe and enduring compulsive overeating that are recognised in the classifications of mental disorders, more recently, the concept of food addiction (FA), defined as an “addiction-like behaviour that develops in association with an excessive, compulsive and dysregulated consumption of energy-dense foods”, has emerged as a relatively new approach to understanding problematic eating behaviours [23,24]. Although controversy persists in how to clearly distinguish the diagnosis of FA from other syndromes, such as eating disorders [25], fixed criteria to assess FA have been established to ensure the homogeneity of results between and across studies worldwide [26]. In this context, most of the current research studies focusing on FA use the Yale Food Addiction Scales (YFASs), which have specifically been designed to measure FA by applying the substance-related and addictive disorders criteria of the DSM to the intake of energy-dense food items (DSM-IV-TR: seven criteria assessed by the YFAS; DSM 5: nine criteria assessed by the YFAS 2.0) [25,26,27]. In effect, similar to addictive drugs, increasing evidence suggests that foods rich in saturated and trans fats, salt and added sugars, which are typical of Western diets, appear to overstimulate neurobiological reward pathways and increase the release of dopamine, thereby triggering FA [28,29,30,31].

Importantly, although previous studies have highlighted high YFAS scores in patients with eating disorders (ED), obesity and/or major depressive disorders (MDD) [32,33,34], the concept of FA in cardiovascular diseases remains largely overlooked. Notably, we were unable to find any prospective studies reporting information on the potential influence of disordered eating behaviours or ED on stroke outcomes. Yet, considering the reported pooled prevalence of FA in the adult population (24%, 95% CI {20; 29}) [35] as well as the negative impact of addictive-like eating behaviours on several markers of metabolic disturbance [36], this research proves important given that the presence of metabolic disturbances has been consistently associated with an increased odds of stroke in the extant literature [37].

Therefore, the aims of this retrospective hospital-based study were to explore the prevalence and severity of FA among stroke survivors and to assess the associations between FA and four critical vascular risk factors for stroke, including hypertension, diabetes, obesity, and dyslipidaemia [38].

## 2. Materials and Methods

### 2.1. Study Procedure and Population

This study was a retrospective analysis of patients consecutively hospitalised for a first stroke event at the Neurovascular Unit of the Bordeaux University Hospital between March and July 2019 and discharged to home. Upon admission to the neurovascular unit, all patients are informed about the potential use of their anonymised clinical, imaging, and biological data for teaching and research purposes, and a non-opposition procedure is applied. In acute care, routine evaluations include neuropsychological assessments and a set of self-reported questionnaires. They are administered to patients who understand French well enough, with no and/or medical history or current medical condition preventing their completion according to the referent neurologist (with a visual disability, severe stroke, a disease affecting the nervous system such as epilepsy or dementia, a severe cognitive impairment, psychiatric diagnosis such as neurodevelopmental or schizophrenia spectrum disorders, and/or severe aphasia) and after obtaining an informed verbal consent. For the present study, the authorisation to use retrospective data from medical electronic records for publication was approved by the Research Ethics Committee of Bordeaux University Hospital (CER-BDX-2022-04) in January 2022 and conducted in accordance with the latest version of the Declaration of Helsinki.

### 2.2. Determination of Food Addiction (FA) in Stroke Patients

In this study, addictive-like eating patterns were only determined using the YFAS 2.0 questionnaire that has been developed by Gearhardt and colleagues [26,39] and validated for French-speaking individuals [27]. Briefly, the YFAS 2.0 is a self-reported questionnaire that is assumed to assess each of the 11 DSM-5 substance use disorders criteria applied to the intake of food items high in saturated fats, refined carbohydrates, and/or sugar and salt (See Supplementary Materials for the YFAS2.0 instruction) plus the associated clinically significant impairment or distress [40]. Although the wider body of the literature focuses primarily on the FA diagnostic scoring option of the scale (i.e., when the participant endorses 2 or more symptoms plus clinically significant impairment or distress), this study also assessed the symptom count scoring option (number of FA symptoms endorsed, without the presence of clinically significant impairment or distress), as well as their proportion across the four domains of addiction symptoms: (1) impaired control; (2) risky use; (3) pharmacological criteria; and (4) social impairment. For individuals endorsing a diagnosis of FA, severity thresholds were also specified (mild: 2–3 symptoms plus impairment or distress; moderate: 4–5 symptoms plus impairment or distress; severe: 6 or more symptoms plus impairment or distress). We complemented our approach to the severity of the phenotype by using a Global Addictive-like eating Profile Severity Index (GAPSI), as explained in statistical analyses Section 2.5.2.

### 2.3. Assessment of Stroke Severity

To evaluate the baseline neurologic outcome and stroke severity in patients, the National Institutes of Health Stroke Scale (NIHSS) was used. The NIHSS is a valid, reliable, and responsive tool assessing domains of (1) consciousness, (2) eye movement, (3) visual fields, (4) facial palsy, (5) motor arm, (6) motor leg, (7) limb ataxia, (8) sensory, (9) language aphasia, (10) speech (dysarthria), and (11) hemi-inattention (neglect). Individual scores from each item are summed, with a minimum and maximum score of 0 and 42, respectively [41].

### 2.4. Key Outcome

This study assessed the association of FA with four of the main vascular risk factors of stroke: obesity; diabetes; hypertension; and dyslipidaemia as independent and cumulative values. BMI was measured using the standard formula (weight (kg)/(height (m^2^)) and categorised patients as underweight (BMI < 18.5 kg/m^2^), healthy (18.5–24.9 kg/m^2^), overweight (25–29.9 kg/m^2^), and obese (≥30 kg/m^2^), according to the World Health Organisation’s criteria for Caucasian populations. Diabetes was diagnosed if the level of haemoglobin A1c (HbA1c) exceeded the cut-point of 48 mmol/mol (6.5%) or if there was a previous medical history of the disease. Patients were considered hypertensive if they presented a medical history of hypertension or if blood pressure exceeded 140/90 mm of mercury (mm Hg). Dyslipidaemia was defined based on fasting serum lipid profile values according to the National Cholesterol Education Program (NCEP) Expert Panel on Detection, Evaluation, and Treatment of High Blood Cholesterol in Adults (ATP III) [42].

### 2.5. Statistical Analyses

#### 2.5.1. Participant Characteristics

Descriptive data were summarised using means (standard deviation (SD)) for continuous variables and frequency for categorical variables. We checked the normality of the key variables of interest by performing the Kolmogorov–Smirnov test and probability plots, and all were not normally distributed. Two-group comparisons for each vascular risk factor (coded as binary indicators), demographic and clinical variables, were performed using univariate analyses for non-normally distributed variables (Mann–Whitney *U* test, Pearson chi-square (χ^2^), or Fisher’s test for independent sample, as appropriate).

#### 2.5.2. Severity of FA

The YFAS2.0 assesses how frequently (from “Never” to “Every day”) in the past year people experienced symptoms related to the difficulties of controlling the intake of energy-dense foods. Gearhardt and colleagues elaborated on a combination of frequency and dichotomous scoring options, where frequency scoring aimed at assessing disordered eating behaviours such as excess consumption or emotional eating, and dichotomous scoring aimed at identifying more severe and problematic eating problems [26]. To determine the severity of the overall addictive-like eating phenotype and take into account the frequency scoring of all the symptom criteria, we computed a Global Addictive-like Eating Profile Index (GAPSI). The items were scored on a four-category ordered scale: 1 = from “Never” to “Less than once a month”; 2 = from “Once a month” to “Two to three times a month”; 3 = from “Once a week” to “Two to three times a week”; and 4 = from “Four to six times a week” to “Every day”. As the evaluation of each symptom encompassed several items, the categorisation was based on the one item endorsing the highest frequency score.

A principal component analysis (PCA) was then applied to all the 11 addiction-related symptoms plus 1 item summarising the associated clinically significant impact (distress and/or impairment), scored on the four-category-ordered scale. The first component was used to build a weighted summary or global severity index for the addictive profile (see Supplementary Table S1). PCA scores reflect the combination of the symptoms weighted according to their contribution to the factor. Higher PCA scores reflect higher individual frequency scores on most of the symptom criteria. Restricted cubic splines (three knots) were used to take into account possible departure from linearity in the case of continuous predictors (age and BMI). The number of predictors was deliberately limited to those known from the literature review and univariate testing in order to conform to the 10–15 events per variable rule of thumb (even if the lowest minority class was 20 positive cases out of 98 in the case of diabetes). The parameters of those models were estimated using ridge (L2) penalisation, whereby the tuning of model hyper-parameters (penalty) was performed using repeated 10-fold cross-validations (25 repetitions). The final solution chosen was the one that minimised the cross-validated log likelihood on the optimal penalty parameter the most.

Parameter estimates corrected for overfitting were obtained using bootstrap resampling (B = 200): for each bootstrap sample, the same regression model was used, and the accuracy measure of each parameter was compared to that found in the original sample, and a final optimism corrected estimate was computed by averaging those apparent biases over all bootstrap iterations. The calibration of each regression model was assessed by comparing predicted probabilities with observed frequencies for the corresponding outcome. The pseudo-R-squared value and Somer’s D index were used to assess the goodness of fit of each model.

#### 2.5.3. Associations Between Cumulative Vascular Risk Factors and Individual Food Addiction (FA) Symptom

Each vascular risk factor was scored, and the sum of vascular risk factors was treated as ordered categorical variables, ranging from 0 to 4 (i.e., the presence of hypertension (score 1), obesity (score 1), diabetes (score 1), and dyslipidaemia (score 1)). Associations between the number of vascular risk factors, the severity of FA symptoms, and symptom counts were then analysed using Spearman’s rank correlation coefficient, which did not assume a normal distribution of the variables. The null hypothesis (H0) was tested, with Spearman’s rank correlation coefficient equal to 0. The alternative hypothesis was that the correlation coefficient was different from 0. The null hypothesis (H0) was rejected at *p* < 0.05 (α = 0.05). To control for age and sex as potential confounders based on our univariate analyses, partial correlations were also performed. In these analyses, given that multicollinearity between age and sex might obscure the computation because of the overlapping correlation, each confounder was analysed separately.

#### 2.5.4. Statistical Software and Thresholds

All statistical tests were performed using R statistical software version 4.1.0 and R package Regression Modeling Strategies (rms) version 6.1-0 (https://CRAN.R-project.org/package=rms, accessed on 24 October 2024) and by considering a fixed type I error rate of 5%.

## 3. Results

### 3.1. Description of the Study Population

Complete data from 101 participants were retrieved from electronic medical records, except for one participant with a missing BMI value. Participant characteristics, captured at baseline and classified according to the presence of vascular risk factors (diabetes, obesity, hypertension, dyslipidaemia), are presented in Table 1. The study sample was predominantly males (60.4%), on average, overweight (BMI: mean (SD), 27.6 (5.77) kg/m^2^), with a mean (SD) age of 62.8 (13.7) years. Among all stroke cases, 86.1% were identified as ischemic stroke patients, 5% as haemorrhagic stroke patients, and 8.9% were diagnosed with transient ischaemic attack (TIA). The mean (SD) NIHSS score was at 2.31 (3.13) points for the overall sample, representing a minor stroke severity as per the stroke scale [43].

### 3.2. Prevalence of Food Addiction (FA) Diagnosis and Symptoms

Table 2 shows the prevalence of patients with a diagnosis of FA and the detailed prevalence of addictive-like eating behaviours by symptoms endorsed. In this study, 5% (*n* = 5) of participants, all with ischemic stroke, endorsed a diagnosis of FA. Interestingly, whereas the endorsement of either clinical distress or impairment is necessary to establish an FA diagnosis, these factors were only experienced by 3.0% and 7.9% of patients, respectively, thereby corresponding with the overall low prevalence of FA in this sample. However, 23.8% of patients screened positive for at least two of the symptom criteria, and 9.0% of patients endorsed at least four symptoms. The most prevalent symptoms (i.e., >10%) were “Inability to cut down” (16.8%), “Use in physically hazardous situations” (15.8%), “Use despite social/interpersonal problems” (12.9%), “Feelings of withdrawal symptoms” (11.9%), and “Tolerance” (10.9%). When combined under specific domains, “Impaired control” was the most prevalent domain, with 25.7% of patients experiencing at least one symptom of this domain.

### 3.3. Food Addiction (FA) Diagnosis, Its Severity, and the Association with Vascular Risk Factors

Table 3 shows the characteristics of patients who met the diagnosis of FA. The mean (SD) age of the patients with FA was 57.0 (13.7) years, with three individuals out of five living with obesity and being females. Four out of five of the patients experienced severe FA, with only one patient suffering from mild FA but living with multi-morbidities, including obesity, hypertension, dyslipidemia and diabetes (Patient 1). The GAPSI score was 2.7 in the patients with mild FA and ranged from 5.2 to 7.2 in those experiencing severe FA.

As shown in Table 4, dyslipidemia was the only vascular risk factor significantly associated with the FA diagnosis *(p* = 0.043, 95% CI [−0.21; 0.01]), with four out of five patients with an FA diagnosis having dyslipidemia, whereas diabetes was present in only one patient. Higher GAPSI scores were also associated with dyslipidemia (*p* = 0.016, 95% CI [−2.16; −0.21]) and diabetes (*p* = 0.038, 95% CI [−1.77; 0.25]) in univariate comparisons. However, GAPSI scores were independently associated with dyslipidemia only (*p* = 0.05; OR = 1.25; 95% CI = 1.00–1.56) (See Supplementary Materials for the detailed regression models with each vascular risk factor).

### 3.4. Associations Between the Food Addiction (FA) Symptom Criteria and Vascular Risk Factor Scores

The correlation coefficients, as well as *p*-values, are presented in Table 5. The relationship between the total number of symptoms endorsed and the number of vascular risk factors was significant only in univariate analyses. When analysing the severity of each specific symptom criterion, there were no significant associations observed with the number of vascular risk factors and most of the FA symptoms, except for (i) Time spent, (ii) Tolerance, and (iii) Use despite adverse emotional or physical consequences. These correlations remained significant after controlling for age and sex.

## 4. Discussion

### 4.1. Principal Findings

To the best of our knowledge, this was the first study to investigate the prevalence of FA and the association of addictive-like eating patterns in the presence of vascular risk factors in a sample of adults with a first-ever mild stroke event. The diagnostic threshold for FA (i.e., two or more symptoms plus clinically significant impairment or distress) was only met by 5% of participants. From this sub-sample group, dyslipidaemia was the only vascular risk factor, which was significantly associated with a diagnosis of FA, and the severity of the addictive profile was independently associated with dyslipidemia only. Concerning the relationship between vascular risk factors and the severity of FA symptoms in the overall sample, significant associations were noted between the number of vascular risk factors and the severity of Time spent, Tolerance, and Use despite adverse consequences symptom criteria, both in univariate and multivariate analyses. However, the number of vascular risk factors and total number of symptoms endorsed were significantly correlated in univariate analyses only.

### 4.2. Prevalence of Food Addiction (FA) Diagnosis and Comparison with Other Studies

Compared with the average prevalence of FA reported in the general French-speaking population at 8.2% [27], our study reported a slightly lower percentage of participants who met the diagnostic threshold for FA at 5%. One potential explanation could pertain to the low endorsement of both clinical distress and impairment (clinical distress: 3%; clinical impairment: 7.9%), either of which is required to establish an FA diagnosis. Based on the YFAS 2.0 scoring instructions, given that clinically significant impairment or distress should be experienced at least twice a week to meet the diagnostic criterion, it is possible that the pre-defined thresholds were too high for some patients, thereby precluding the diagnosis of FA. In addition, whereas clinical impairment or distress has more often been reported among young females [44,45] and people living with obesity [46,47], our overall study sample was predominantly males (60.4%), in their later adulthood stages at the mean (SD) age of 62.8 (13.7) years, and with a mean (SD) BMI representing overweight at 27.6 (5.77) kg/m^2^. In this context, the demographics of our overall study sample may have also precluded a diagnosis of FA in many participants. This finding could, in effect, be substantiated by the fact that our subsample of patients with an FA diagnosis was, on average, 5 years younger than the overall sample, of whom two were females with severe FA (Table 3). However, given that 23.8% of participants screened positive for at least two FA symptoms, the use of the YFAS 2.0 questionnaire solely as a diagnostic scoring option appears reductive in the context of stroke, considering that the types and number of symptoms with and without the endorsement of clinical distress or impairment criterion could be informative for research and treatment [48], as discussed in Section 4.3.

### 4.3. Endorsement Rates for Food Addiction (FA) Symptom Criteria in the Overall Sample

Overall, 38.6% of the patients screened positive for at least one of the 11 FA symptom criteria (i.e., without clinical impairment or distress). Consistent with other studies [45,49,50], the most frequent symptom endorsed by the current sample was “Inability to cut down”. One potential explanation underpinning this common finding across cohorts may pertain to the acquired benefits from the ubiquity of low-cost, energy-dense, and palatable ultra-processed foods (UPFs) and the abundance of external cues recalling triggers of snacking behaviours [51]. Although UPFs form a highly heterogeneous food category and also include low-calorie products, products “fortified” with beneficial nutrients or frozen meals [52], the majority of UPFs are high in energy density, salts, added sugars, saturated and trans fats, as well as additives [53]. Mechanistically, preclinical and human studies have demonstrated that a repeated intake of UPFs triggers addiction-like biological (e.g., dopaminergic sensitisation) and behavioural responses (e.g., tolerance, withdrawal, use despite consequences) through the pharmacokinetic properties of rewarding ingredients (e.g., fatty–salty and fatty–sugary) that are rapidly absorbed by the system [54]. However, a complementary assessment of dietary intake, allowing for a detailed analysis regarding the amount of high-fat or high-sugar foods consumed, would be needed to confirm these explanations.

### 4.4. Association Between Food Addiction (FA) Diagnosis and Vascular Risk Factors

In this case series (*n* = 5 with an FA diagnosis), there were no robust associations between FA diagnosis and diabetes, obesity, and/or hypertension. Clinically, given that it has been shown that type 2 diabetic patients with an FA diagnosis are mostly in the age range of 20–29 years [55], it is possible that associations between an FA diagnosis and vascular risk factors may be stronger in younger stroke survivors. However, more needs to be understood about developmental changes in healthy and unhealthy food preferences [56], as several studies have shown age-related effects on central regulation [57,58]. Compared to older adults, younger individuals have been shown to be more motivated by food preferences, to experience greater food cravings for unhealthy foods, and to have increased striatal sensitivity to food stimuli, all of which corresponded with an imbalanced development of the prefrontal cortex and limbic system [59]. Importantly, besides the potential long-term neurological repercussions in younger individuals [60], these connections between brain health and dietary intake also parallel the premature onset of vascular risk factors for cardiovascular diseases [61,62,63]. To this end, the lack of significant relationship in our study sample could be due to the fact that the patients of this study belonged primarily to Generation X and Boomers II cohorts and, therefore, might have been less exposed to calorie-dense environments in their earlier stages of life.

Nevertheless, whilst the possibility of a type I error cannot be negated with regard to the limited sample size, FA and dyslipidaemia were significantly associated in this study sample. Moreover, if dyslipidaemia was the only vascular risk factor significantly associated with FA in this study sample, there were more patients who suffered from dyslipidaemia (*n* = 34) compared with diabetes (*n* = 21). From a cultural perspective, considering that older French adults are more likely to regularly consume higher quantities of high-fat foods (e.g., butter, cheeses, sausages, and other charcuterie) than high-sugar foods as part of their daily diets [64,65], it may be possible that the association between FA and dyslipidaemia is, thus, more significant in this study sample. However, despite not meeting the criteria for an FA diagnosis, similar results were reported by Nelder and colleagues in the general Newfoundland population, whereby YFAS symptoms were significantly associated with triglyceride levels and inversely associated with high-density lipoproteins (HDL) levels in a larger study sample of healthy adults [36]. Mechanistically, the associations between FA and dyslipidaemia could be supported by preclinical studies, whereby prolonged high-fat intake in rodents has been shown to reduce mesolimbic dopamine (DA) function and nucleus accumbens (NAc) DA overflow, besides modulating striatal neuroadaptive responses and blunting DA reuptake in the NAc [66,67,68,69]. Importantly, although evidence for causality remains enigmatic, these observations could be potentially problematic considering the co-existence of diabetes, obesity, and hypertension with dyslipidaemia [70,71,72]. However, given that our own results indicated no significant correlation between the number of vascular risk factors and symptom counts in controlled analyses and no significant association between the number of vascular risk factors and most FA symptoms in multivariate analyses (except for Time spent, Tolerance, and Use despite adverse consequences), larger and longer-term studies are warranted to improve understanding of the impact of such eating behaviours on health, as well as the direction of association between FA and vascular risk factors.

### 4.5. Strengths and Limitations of This Study

The sample size analysed was rather small, thereby reducing the statistical power to detect potential significant associations, and even with multivariable adjustments, measurement error-related or residual confounding cannot be excluded in our exploratory observational study design. Moreover, due to the cross-sectional design of this study, our statistical results inherently possess limitations that preclude causal interpretations.

The final sample lacked age and sex diversity. As previously discussed, these demographic factors are of special interest given that the prevalence of FA is not only higher in young than older population but also more elevated among females than males. In the present study, the age distribution is as expected for a stroke population, especially of minor or moderate severity. While the men-to-women distribution was not representative of the typical sex ratio of stroke patients, it corresponded to that of a non-severe stroke population. In addition, whereas stroke and FA association may differ by race, ethnicity, genetic composition and physical activity level [9,73,74], our study did not measure these potential key confounders, thereby impacting the generalisability of the findings. Indeed, beyond the numerous studies that highlighted a significant genetic component in the occurrence of stroke, a dopamine genetic risk has been associated with FA [75,76,77,78]. Blacks, Hispanics, and those with sedentary behaviours have also been shown to have an increased risk of both stroke and FA [79,80,81].

Regarding the evaluation of at-risk lifestyle behaviours among the stroke population, the possibility of recall and social desirability biases from the self-reported YFAS 2.0 questionnaire cannot be excluded. Therefore, future studies with larger and more diverse samples, including a complimentary assessment of dietary intake that would ensure a detailed analysis regarding the amount of high-fat or high-sugar foods consumed, are needed to draw firm conclusions.

## 5. Conclusions

From the evaluation of the extant literature, our study findings emphasise a research gap pertaining to disordered eating patterns in people with stroke. Our study highlighted the potential importance of considering addictive-like eating behaviours as part of the management of stroke patients. Whereas there is still some uncertainty from the substantive Cochrane Review regarding the benefits of adopting healthy dietary patterns such as a Mediterranean-style diet for stroke secondary prevention [19], we believe that administering the YFAS 2.0 questionnaire as part of routine evaluations could be used to identify patients with specific unmet needs and unhealthy eating patterns in their care pathways. In this context, to optimise the secondary prevention of stroke, it is important to recognise addictive-like eating behaviours as complex and multifaceted, thus requiring a multidisciplinary team approach. For example, the collaboration between not only neurologists and dietitians but also with addictologists/psychiatrists and psychologists specialised in disordered eating and associated disorders may be conducive to reducing the determinants of such compulsive overeating behaviours. Through a multidisciplinary lens, future prospective studies should prioritise the investigation of FA and its symptoms in larger and more diverse samples of stroke patients so as to better guide clinical practice and achieve precision medicine.

## Figures and Tables

**Table 1:** Characteristics of all study participants (*n* = 101) and univariate between-group comparisons.

		Hypertension			Diabetes			Dyslipidaemia			Obesity		
Characteristics	Overall	HTN− (*n* = 49)	HTN+ (*n* = 52)	*p*-Value, (ES)	DIA− (*n* = 80)	DIA+ (*n* = 21)	*p*-Value, (ES)	DYS− (*n* = 67)	DYS+ (*n* = 34)	*p*-Value, (ES)	OB− (*n* = 71)	OB+ (*n* = 29)	*p*-Value, (ES)
**Sex, % men (*n*)**	60.40 (61)	61.20 (30)	59.60 (31)	0.870 (0.387)	56.30 (45)	76.20 (16)	0.096 (0.165)	56.70 (38)	67.60 (23)	0.288 (0.106)	63.40 (45)	51.70 (15)	0.320 (−0.099)
**Age, years (mean (SD))**	62.80 (13.7)	56.30 (14.0)	68.90 (10.1)	**<0.001** **(−0.461)**	61.70 (14.7)	66.80 (7.80)	0.215 (−0.124)	60. 60 (14.6)	67.10 (10.6)	**0.032** **(−0.214)**	61.40 (14.8)	66.40 (9.87)	0.305 (−0.102)
**NIHSS (mean (SD))**	2.31 (3.13)	1.84 (2.94)	2.75 (3.30)	**0.014** **(0.370)**	2.34 (3.42)	2.19 (1.66)	0.151 (0.253)	2.06 (2.48)	2.79 (4.13)	0.470 (0.403)	2.13 (3.08)	2.83 (3.29)	0.063 (0.381)
BMI*, kg/m^2^ (mean (SD)) (*n* = 100)	27.60 (5.77)	26.00 (5.02)	28.90 (6.22)	**0.009** **(−0.260)**	27.10 (5.52)	29.00 (6.75)	**0.034** **(−0.212)**	26.60 (5.07)	29.20 (6.86)	**<0.001** **(0.807)**	24.60 (2.96)	34.50 (5.01)	**<0.001** **(−0.777)**
**Comorbidities**													
**Obesity, % (*n*) (*n* = 100)**	29.00 (29)	18.40 (9)	39.20 (20)	**0.022** **(0.629)**	26.60 (21)	38.10 (8)	0.310 (0.572)	23.90 (16)	39.40 (13)	0.110 (0.886)	-	-	-
**Dyslipidaemia, % (*n*)**	33.70 (34)	14.30 (7)	51.90 (27)	**<0.001** **(0.452)**	26.30 (21)	61.90 (13)	**0.002** **(0.750)**	-	-	-	28.20 (20)	44.80 (13)	0.110 (0.773)
**Diabetes, % (*n*)**	20.80 (21)	6.10 (3)	34.60 (18)	**<0.001** **(0.376)**	-	-	-	11.90 (8)	38.20 (13)	**0.002** **(0.763)**	18.30 (13)	27.60 (8)	0.301 (0.763)
**Hypertension, % (*n*)**	51.50 (52)	-	-	-	42.50 (34)	85.70 (18)	**<0.001** **(0.654)**	37.30 (25)	79.40 (27)	**<0.001** **(0.950**)	43.70 (31)	69.0 (20)	**0.022** **(0.747)**

HTN, DIA, DYS, OB−: patients without hypertension, diabetes, dyslipidemia, obesity; HTN, DIA, DYS, OB+: patients with hypertension, diabetes, dyslipidemia, obesity; ES: effect size; BMI: body mass index; BMI* *n* = 100; NIHSS: National Institute of Health Stroke Scale; SD: Standard deviation. Bold figures indicate significance at *p* < 0.05.

**Table 2:** Prevalence of Food Addiction (FA) diagnosis and detailed symptom count (*n* = 101).

	Prevalence, % (*n*)
**FA diagnosis**	
Two or more symptoms plus clinically significant distress or impairment	5.0 (5)
**Symptom criteria**	
(1) Inability to cut down	16.8 (17)
(2) Physically hazardous situations	15.8 (16)
(3) Interpersonal problems	12.9 (13)
(4) Withdrawal	11.9 (12)
(5) Time spent	10.9 (11)
(6) Tolerance	8.9 (9)
(7) Loss of control	6.9 (7)
(8) Adverse consequences	5.9 (6)
(9) Craving	5.9 (6)
(10) Impaired daily functioning	5.0 (5)
(11) Activities given up	4.0 (4)
**Clinical significance**	
Distress	3.0 (3)
Impairment	7.9 (8)
**Domains (symptom criteria)**	
Impaired control (symptoms 1, 5, 7, 9)	25.7 (26)
Risky use (symptoms 2, 8)	18.8 (19)
Pharmacological criteria (symptoms 4, 6)	16.8 (17)
Social impairment (symptoms 3, 10, 11)	16.8 (17)
**Symptom count**	
No symptom	61.4 (62)
One symptom endorsed	14.9 (15)
Two symptoms endorsed	5.9 (6)
Three symptoms endorsed	8.9 (9)
Four symptoms endorsed	2.0 (2)
Five symptoms endorsed	2.0 (2)
Six symptoms endorsed	2.0 (2)
Seven symptoms endorsed	2.0 (2)

**Table 3:** Characteristics of patients with a Food Addiction (FA) diagnosis.

Characteristics	Patient 1	Patient 2	Patient 3	Patient 4	Patient 5
Sex	Male	Female	Male	Female	Female
Age, y	63	38	64	48	72
NIHSS	3	7	11	1	2
Weight status	Obese	Healthy	Obese	Healthy	Obese
BMI, kg/m^2^	31.6	24.4	40.8	21.3	37.2
Hypertension	Yes	No	No	No	Yes
Diabetes	Yes	No	No	No	No
Dyslipidaemia	Yes	Yes	Yes	No	Yes
FA severity	Mild	Severe	Severe	Severe	Severe
GAPSI	2.7	7.2	6.7	5.2	11.2
**YFAS symptom criteria:**					
1. Substance taken in larger amounts and for longer periods than intended		x		x	
2. Persistent desire or repeated unsuccessful attempts to quit		x	x	x	
3. Much time/activity to obtain, use, recover	x		x	x	x
4. Important social, occupational, or recreational activities given up or reduced	x		x		x
5. Use continues despite knowledge of adverse consequences			x		x
6. Tolerance		x	x	x	
7. Characteristic withdrawal symptoms; substance taken to relieve withdrawal	x	x	x		x
8. Continued use despite social or interpersonal problems		x		x	x
9. Failure to fulfil major role obligations (e.g., work, school, home)			x		x
10. Use in physically hazardous situations		x			x
11. Craving or a strong desire or urge to use		x		x	x
12. Use causes clinically significant impairment or distress	x	x	x	x	x

NIHSS: National Institute of Health Stroke Scale; BMI: Body mass index; GAPSI: Global Addictive-like Eating Profile Severity Index.

**Table 4:** Associations between Food Addiction diagnosis by vascular stroke risk factor and the Global Addictive-like Eating Profile Severity Index (GAPSI).

		Vascular Risk Factors															
		Hypertension				Diabetes				Dyslipidaemia				Obesity			
Characteristics	Overall	HTN− (*n* = 49)	HTN+ (*n* = 52)	*p*- Value	95% CI	DIA− (*n* = 80)	DIA+ (*n* = 21)	*p*- Value	95% CI	DYS− (*n* = 67)	DYS+ (*n* = 34)	*p*- Value	95% CI	OB− (*n* = 71)	OB+ (*n* = 29)	*p*- Value	95% CI
FA diagnosis, % (*n*)	5 (5)	40 (2)	60 (3)	0.671	[−0.10; 0.07]	80 (4)	20 (1)	0.964	[−0.10; 0.11]	20 (1)	80 (4)	**0.043**	[−0.21; 0.01]	40 (2)	60 (3)	0.151	[−0.19; 0.04]
GAPSI, Mean (SD)	−3.05^−15^ (2.23)	−0.18	0.17	0.150	[−1.23; 0.53]	−0.16	0.6	**0.038**	[−1.77; 0.25]	−0.37	0.72	**0.016**	[−2.16; −0.21]	−0.31	0.7	0.090	[−2.21; 0.19]
		−2.25	−2.23			−2.28	−1.96			−1.77	−2.83			−1.81	−2.96		

GAPSI: Global Addictive-like Eating Profile Severity Index; 95% CI: 95% confidence interval. Bold figures indicate significance at *p* < 0.05.

**Table 5:** Correlations between the number of vascular risk factors (0–4), the symptom count, and the severity of each symptom criterion. Values presented are correlation coefficients, ρ (rho), and *p*-value.

	Association with Number of Vascular Risk Factors		
	Unadjusted, ρ (*p*-Value)	Adjusted for Age, ρ (*p*-Value)	Adjusted for Sex, ρ (*p*-Value)
Number of symptoms endorsed	**0.265 (0.007)**	0.194 (0.055)	0.174 (0.083)
Severity of symptoms criteria			
(1) Inability to cut down	0.028 (0.782)	−0.009 (0.928)	0.023 (0.817)
(2) Physically hazardous situations	−0.042 (0.680)	−0.004 (0.968)	−0054 (0.597)
(3) Interpersonal problems	−0.113 (0.261)	−0.057 (0.575)	−0.116 (0.249)
(4) Withdrawal	0.087 (0.387)	0.021 (0.833)	0.051 (0.616)
(5) Time spent	**0.219 (0.028)**	**0.212 (0.035)**	**0.236 (0.018)**
(6) Tolerance	**0.260 (0.009)**	**0.343 (<0.001)**	**0.319 (0.001)**
(7) Loss of control	0.132 (0.189)	0.041 (0.684)	0.021 (0.837)
(8) Use despite adverse consequences	**0.230 (0.020)**	**0.251 (0.012)**	**0.214 (0.033)**
(9) Craving	0.072 (0.472)	0.094 (0.357)	0.059 (0.558)
(10) Impaired daily functioning	−0.065 (0.518)	0.010 (0.922)	−0.024 (0.813)
(11) Activities given up	0.081 (0.419)	0.108 (0.287)	0.103 (0.307)

## Data Availability

The data presented in this study are available upon reasonable request from the corresponding author. The data are not publicly available due to specifications from the study’s sponsor.

## Supplementary Materials

The following supporting information can be downloaded at: https://www.mdpi.com/article/10.3390/nu16244327/s1, Table S1. Principal component analysis on the YFAS2.0 items used to compute the Global Addictive-like eating Profile Severity Index (GAPSI). Figure S1. Predicted values of Pr(Dyslipidemia).
