# Supplementary material for: Food Addiction: Prevalence, Severity, and Impact on Vascular Stroke Risk Factors in a French Hospital-Based Sample
Source: Nutrients. 2024 Dec 15;16(24):4327. doi: 10.3390/nu16244327 (PMC11676678; doi:10.3390/nu16244327)

SUPPLEMENTARY MATERIAL

Food Addiction: Prevalence, Severity and Impact on Vascular Stroke Risk Factors in a French Hospital-Based Sample

Bibi Aliya Seelarbokus [1,*], Yolaine Rabat [1,*], Christophe Lalanne [2], Igor Sibon [1,3,#] and Sylvie Berthoz [1,4]

[1] Univ. Bordeaux, Institute for Cognitive and Integrative Neuroscience Aquitaine (INCIA), French National Centre for Scientific Research (CNRS), UMR5287, 33000 Bordeaux, France;
[2] University Paris Cité, Paris, France
[3] Neurovascular Unit, Bordeaux University Hospital, 33000 Bordeaux, France;
[4] Department of Psychiatry of Adolescents and Young Adults, Institut Mutualiste Montsouris, Paris, France
*Authors contributed equally
[#] Corresponding author: igor.sibon@chu-bordeaux.fr

Supplemental Information

Instruction for completing the Yale Food Addiction Scale Version 2.0

Supplemental Results

Principal component analysis on the YFAS2.0 addiction-related items used to compute the Global Addictive-like eating Profile Severity Index (GAPSI)

Association between the global severity of the addictive profile with each risk factors

Predicted values of Pr(Dyslipidemia)

Instruction for completing the Yale Food Addiction Scale Version 2.0

"This survey asks about your eating habits in the past year. People sometimes have difficulty controlling how much they eat of certain foods such as:

- Sweets like ice cream, chocolate, doughnuts, cookies, cake, candy
- Starches like white bread, rolls, pasta, and rice
- Salty snacks like chips, pretzels, and crackers
- Fatty foods like steak, bacon, hamburgers, cheeseburgers, pizza, and French fries
- Sugary drinks like soda pop, lemonade, sports drinks, and energy drinks

When the following questions ask about "CERTAIN FOODS" please think of ANY foods or beverages similar to those listed in the food or beverage groups above or ANY OTHER foods you have had difficulty with in the past year".

Supplementary Table 1. Principal component analysis on the YFAS2.0 items used to compute the Global Addictive-like eating Profile Severity Index (GAPSI)

GAPSI scores	Cor (1st axis)	Contribution (%)
(1) Persistent desire or unsuccessful efforts to cut down or control consumption of certain foods.	**0.715**	**10.36**
(3) Continued use despite social or interpersonal problems.	**0.696**	**9.823**
(8) Continued use despite physical or psychological problems.	**0.695**	**9.779**
(5) Considerable time spent to obtain, consume, or recover from effects of food.	**0.689**	**9.62**
(4) Withdrawal.	**0.679**	**9.334**
(9) Craving.	**0.667**	**9.011**
(11) Giving up important social, occupational, or recreational activities	**0.64**	**8.296**
(10) Failure to fulfill major role obligations.	**0.639**	**8.278**
(2) Use in physically hazardous situations.	**0.629**	**8.021**
Clinical significance.	**0.59**	**7.045**
(6) Tolerance.	**0.524**	**5.576**
(7) Consumed in larger quantities or over a longer period than intended.	**0.489**	**4.855**

Supplementary Regression Models

			DYSLIPIDEMIA				
Model 1a. adjusted for Diabetes:							
Predictor	***Estimate***	***SE***	***Z***	***p***	***Penalty-scale***	***OR***	***95%CI***
Sex:	-0.64	0.51	-1.25	0.21	0.71	0.53	0.19-1.44
Age linear:	0.04	0.04	1.06	0.29	13.72	1.04	0.97-1.12
Age quadratic:	0.01	0.04	0.13	0.9	10.36		
BMI linear:	-0.02	0.09	-0.17	0.87	5.81	0.98	0.82-1.18
BMI quadratic:	0.09	0.11	0.77	0.44	4.86		
Diabetes:	1.15	0.55	2.08	0.04	0.41	3.15	1.07-9.27
GAPSI	0.18	0.11	1.6	0.11	2.24	1.2	0.96-1.49

Goodness-of-fit indices	LR Chi^2	Pr(>chi^2)	Penalty	R^2	Brier	C
	21.43	0	1.06	0.27	0.18	0.78

DYSLIPIDEMIA

Model 1b. unadjusted for Diabetes:							
Predictor	***Estimate***	***SE***	***Z***	***p***	***Penalty-scale***	***Odds Ratio***	***95%CI***
Sex	-0.8	0.5	-1.61	0.11	0.71	0.45	0.17-1.19
Age linear	0.06	0.04	1.54	0.12	13.72	1.06	0.98-1.14
Age quadratic	-0.01	0.04	-0.25	0.81	10.36		
BMI linear	-0.02	0.09	-0.18	0.85	5.81	0.98	0.82-1.18
BMI quadratic	0.09	0.11	0.81	0.42	4.86		
GAPSI	0.22	0.11	1.94	0.05	2.24	1.25	1.00-1.56
Goodness-of-fit indices	LR Chi²	Pr(>chi²)	Penalty	R^2	Brier	C	
	17.33	0	1.37	0.21	0.19	0.73	

DIABETES

Model 2							
Predictor	***Estimate***	***SE***	***Z***	***p***	***Penalty-scale***	***OR***	***95%CI***
Sex:	-0.78	0.64	-1.22	0.22	0.5	0.46	0.13-1.61
Age linear:	0.11	0.05	1.97	0.05	9.7	1.11	1.00-1.24
Age quadratic:	-0.1	0.06	-1.74	0.08	7.32		
BMI linear:	0.01	0.12	0.1	0.92	4.11	1.01	0.80-1.28
BMI quadratic:	0	0.14	0.03	0.98	3.43		
Dyslipidemia:	1.16	0.55	2.1	0.04	0.33	3.19	1.08-9.42
GAPSI	0.1	0.12	0.89	0.37	1.58	1.11	0.88-1.39
Goodness-of-fit indices	LR Chi²	Pr(>chi²)	Penalty	R^2	Brier	C	
	19.58	0	1.95	0.25	0.14	0.82	

OBESITY

**Model 3:**							
Predictor	***Estimate***	***SE***	***Z***	***p***	***Penalty-scale***	***OR***	***95%CI***
Sex:	0.6	0.49	1.22	0.22	0.39	1.82	0.69-4.78
Age linear:	0.11	0.05	2.34	0.02	7.52	1.11	1.02-1.22
Age quadratic:	-0.1	0.05	-1.95	0.05	5.67		
GAPSI	0.2	0.11	1.89	0.06	1.23	1.23	0.99-1.52

Goodness-of-fit indices	LR Chi²	Pr(>chi²)	Penalty	R^2	Brier	C
	13.07	0.01	1.11	0.16	0.18	0.7

HYPERTENSION

**Model 4:**							
Predictor	***Estimate***	***SE***	***Z***	***p***	***Penalty-scale***	***OR***	***95%CI***
Sex:	-0.29	0.49	-0.6	0.55	0.71	0.74	0.29-1.95
Age linear:	0.08	0.04	2.03	0.04	13.72	1.08	1.00-1.16
Age quadratic:	0.02	0.04	0.34	0.74	10.36		
BMI linear:	0.08	0.09	0.89	0.37	5.81	1.09	0.91-1.30
BMI quadratic:	0	0.11	0	1	4.86		
GAPSI :	0.07	0.12	0.56	0.58	2.24	1.07	0.85-1.34

Goodness-of-fit indices	LR Chi²	Pr(>chi²)	Penalty	R^2	Brier	C
	30.11	<0.001	1.37	0.33	0.19	0.79

Suppl. Figure 1. Predicted values of Pr(Dyslipidemia)

Each panel represents the expected values predicted by a multivariate logistic regression model, adjusted for sex, age, BMI, and GAPSI score depending on the predictor of interest. In the case of discrete predictor, values are expressed as frequency while for continuous predictors expected values are on the log-odds scale on the y-axis. The chi-squared statistic reflects the main effect of the predictor (Wald test). Shaded bands (continuous predictors) and error bars (discrete predictor) represent 95% confidence intervals. Note that BMI and age are modelled using cubic splines, which explains the non-linearity of the fitted curve.

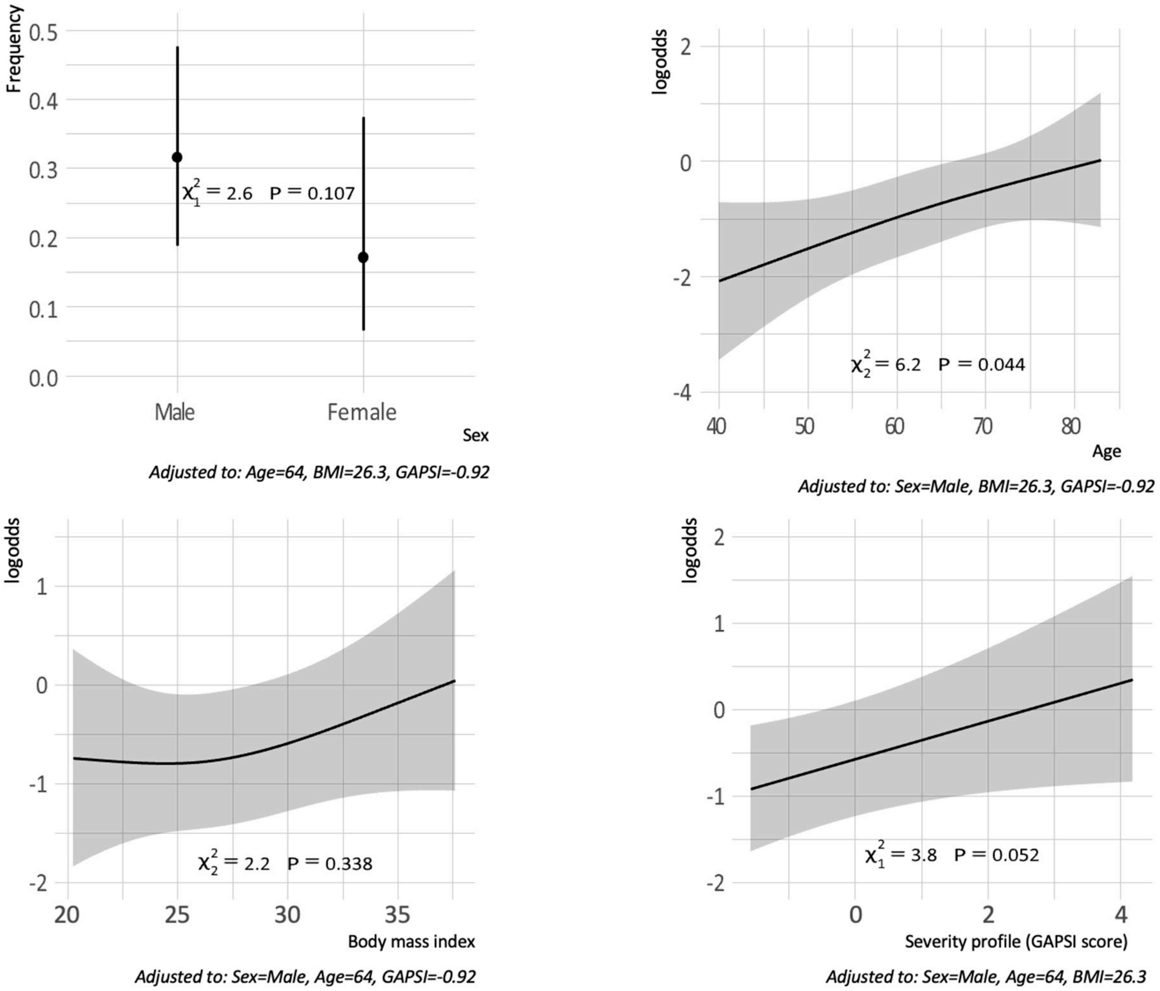

Supplement: Supplementary file 1 [file nutrients-16-04327-s001.zip › nutrients-3363261-supplementary.pdf]
